# Assessment of Osteoporosis in Injured Older Women Admitted to a Safety-Net Level One Trauma Center: A Unique Opportunity to Fulfill an Unmet Need

**DOI:** 10.1155/2017/4658050

**Published:** 2017-11-06

**Authors:** Elisabeth S. Young, May J. Reed, Tam N. Pham, Joel A. Gross, Lisa A. Taitsman, Stephen J. Kaplan

**Affiliations:** ^1^University of Hawaii College of Medicine, Honolulu, HI, USA; ^2^Division of Geriatrics and Gerontology, Department of Medicine, Harborview Medical Center, University of Washington, Seattle, WA, USA; ^3^Department of Surgery, Harborview Medical Center, University of Washington, Seattle, WA, USA; ^4^Department of Radiology, Harborview Medical Center, University of Washington, Seattle, WA, USA; ^5^Department of Orthopedics and Sports Medicine, Harborview Medical Center, University of Washington, Seattle, WA, USA; ^6^Section of General, Thoracic, and Vascular Surgery, Department of Surgery, Virginia Mason Medical Center, Seattle, WA, USA

## Abstract

**Background:**

Older trauma patients often undergo computed tomography (CT) as part of the initial work-up. CT imaging can also be used opportunistically to measure bone density and assess osteoporosis.

**Methods:**

In this retrospective cohort study, osteoporosis was ascertained from admission CT scans in women aged ≥65 admitted to the ICU for traumatic injury during a 3-year period at a single, safety-net, level 1 trauma center. Osteoporosis was defined by established CT-based criteria of average L1 vertebral body Hounsfield units <110. Evidence of diagnosis and/or treatment of osteoporosis was the primary outcome.

**Results:**

The study cohort consisted of 215 women over a 3-year study period, of which 101 (47%) had evidence of osteoporosis by CT scan criteria. There were no differences in injury severity score, hospital length of stay, cost, or discharge disposition between groups with and without evidence of osteoporosis. Only 55 (59%) of the 94 patients with osteoporosis who survived to discharge had a documented osteoporosis diagnosis and/or corresponding evaluation/treatment plan.

**Conclusion:**

Nearly half of older women admitted with traumatic injuries had underlying osteoporosis, but 41% had neither clinical recognition of this finding nor a treatment plan for osteoporosis. Admission for traumatic injury is an opportunity to assess osteoporosis, initiate appropriate intervention, and coordinate follow-up care. Trauma and acute care teams should consider assessment of osteoporosis in women who undergo CT imaging and provide a bridge to outpatient services.

## 1. Introduction

Adults aged 65 and older constitute over 25% of trauma related admissions and are the fastest growing trauma patient population, with a significant proportion sustaining fractures [1]. Osteoporotic fractures, particularly hip fracture, are a significant cause of morbidity and mortality in these patients [2]. Older women are particularly vulnerable to osteoporosis as declining estrogen contributes to an increased rate of bone loss and thus reduced bone mineral density (BMD) that contributes to fracture risk. As the population ages, osteoporosis is a growing individual and public health concern with more than 40 million Americans at risk for this diagnosis [2–4]. The lifetime risk of developing a fracture in patients with underlying osteoporosis is estimated to be up to 50% in women and 20% in men [2].

Over 80 million CT scans are performed annually in the United States and many of these scans are performed on older patients who have undiagnosed chronic conditions. In the routine evaluation of patients with traumatic injury, thorough imaging via computed tomography (CT) is often obtained [5]. Nevertheless, the implications of routine opportunistic utilization of CT scans for the assessment of osteoporosis in acute care settings have not been previously evaluated. Aside from the immediate needs of addressing traumatic injury, harnessing the diagnostic power of routine evaluations in acute care settings may provide significant health and cost benefits.

This repurposing of diagnostic imaging is not a new idea. CT-derived BMD is an established method to identify chronic bone loss, diagnose vertebral fractures, and improve reliability of BMD estimates in patients with aortic calcification [6]. Others have reported diagnosing osteopenia and osteoporosis with CT scans ordered for other reasons and noted substantial opportunities for savings with respect to obviating the expense of additional imaging and the cost of preventable fractures [7, 8].

The use of computed tomography to evaluate bone mineral density has been broadly described, although it has not been widely accepted as a gold standard for routine outpatient screening. Specifically, Pickhardt et al. compared CT-derived BMD to Dual Energy X-ray Absorptiometry (DXA) measures in over 2000 paired comparisons and found highly predictive values for osteoporosis diagnosis (area under the curve [AUC] = 0.83, 95% CI 0.81–0.85) [8]. Subsequent authors have independently demonstrated significant predictive values and correlations between DXA and CT [7, 9–11].

The US Preventive Service Task Force (USPSTF) recommends that all women over 65 years of age should be screened for osteoporosis and that women below 65 years of age should be tested in the presence of additional risk fractures, such as a fragility fracture [2, 3]. Despite these guidelines, many older women do not undergo formal BMD evaluation, even in the context of falls or known fall risk [1, 2].

The purpose of this study was to assess the prevalence of osteoporosis by opportunistic CT imaging in older adult women admitted for trauma, a high-risk population, and measure recognition of this chronic disease by acute care providers in a safety-net hospital.

## 2. Methods

### 2.1. Study Design and Participants

**Figure 1:**
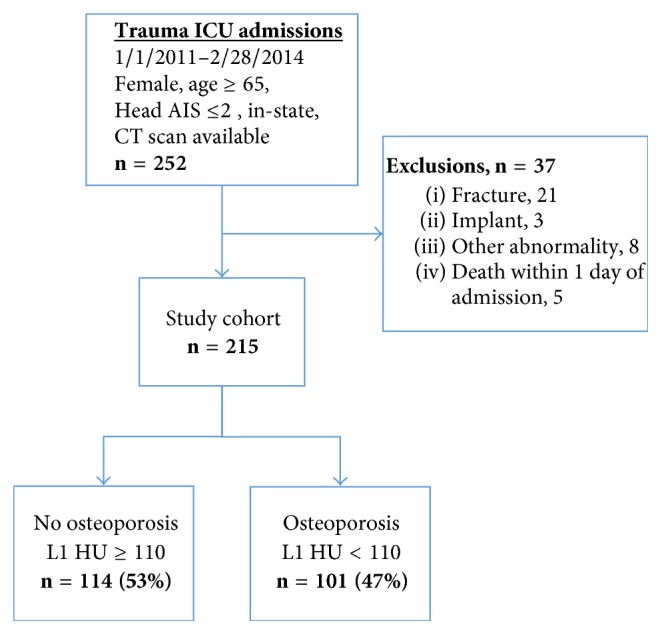
Flow diagram of study patients, showing inclusion and exclusion criteria, grouping of study cohort.

This retrospective cohort study included all women of age ≥ 65 who were in-state residents, sustained traumatic injury without serious head injury (maximum head abbreviated injury severity [AIS] score < 3), had CT imaging of L1 within 7 days of admission, and were admitted to the intensive care unit (ICU) at a safety-net, level one trauma center from January 2011 to February 2014. Our target population was chosen because women are a traditionally at-risk population, with female to male 4 : 1 prevalence of osteoporosis in the US [2]. We also restricted our analysis to patients admitted to the ICU because severely injured patients were more likely to undergo truncal CT evaluation on admission. Inclusion of only in-state residents allowed for readmissions and mortality linkage using state registries as described below. Women who died within 24 hours of admission or whose L1 imaging was inadequate for analysis were excluded (Figure 1).

### 2.2. Study Setting

This study was conducted at a level one trauma center, which serves the surrounding metropolitan area, state, and surrounding four-state region. In addition to being the only level one adult trauma, pediatric trauma, and burn center in the state, the facility also serves as the state's main safety-net hospital. Fifteen percent of the state population is ≥65 years old; 12% of the population is living in poverty (income below 100% of the federal poverty line). Sixty-two percent of patients visiting this hospital qualify for Medicaid or premium subsidies under the state Health Insurance Marketplace. Nearly 50% of the service population are members of racial and/or ethnic minorities; more than 8% are non-English speaking; and more than 2% are indigents without third-party coverage.

### 2.3. Definition of Osteoporosis by CT

Patients were divided into groups by presence or absence of osteoporosis, which was defined as average vertebral body Hounsfield units (HU) < 110 (90% specificity) as described by Pickard et al., 2013 [7, 8, 10, 11].

### 2.4. Covariates

Patient data, injury details, and clinical measures were queried through the state trauma registry. Ground-level falls were determined according to ICD-9 E codes (E880.1, E884.2, E884.3, E884.4, E884.6, E885.9, E888.1, and E888.8). To determine outcomes after trauma, the registry was linked to the Comprehensive Hospital Abstract Reporting System (CHARS), a statewide database that contains hospital admission information. Only the first nonelective readmission after the index trauma hospitalization was included (identified by categorization in CHARS). Trauma Registry and CHARS datasets were further linked to the Washington State Death Registry to assess 30-day and 1-year mortality.

### 2.5. Evidence of Osteoporosis Recognition and Treatment by Inpatient Providers

Recognition of osteoporosis was ascertained from problem list; patient education materials; discharge summary; or treatment of osteoporosis (calcium, vitamin D, bisphosphonate, teriparatide, or denosumab) that was abstracted from the discharge medication list.

### 2.6. Image Analysis Protocol

In the sagittal midline plane, the L1 vertebral body was identified by locating the superior aspect of the sacrum and labeling the immediately superior vertebral body as L5. Identification of L1 was confirmed by absence of ribs at that level. T12 was utilized if L1 was excluded due to Genant Grade II or III compression fracture, neoplastic lesion, hemangioma, or any compromising abnormality that resulted in nonhomogenous bone. L2 was utilized if T12 required exclusion. The most superior axial plane of the chosen vertebral body, which minimized presence of cortical bone and excluded comprising abnormalities, was chosen. Vertebral BMD was assessed by placing a single elliptical region of interest (ROI) 100–120 mm^2^ on the central part of the vertebral body excluding cortical bone, sclerotic bone, or fracture lines. Average HU measurement and SD from the selected vertebra was recorded. Intra- and Interrater reliability of HU measurements was confirmed using intraclass correlation coefficients utilizing the first 32 patients included in the study. The images were analyzed separately by a trauma radiologist (J. A. G.), a research scholar and surgical resident (S. J. K.), and a medical student (E. S. Y.). Intraclass correlation coefficients were 0.98 [95% Cl 0.96–0.99] for HU calculation and 0.99 [95% Cl 0.993–0.999] for axial image selection. All three evaluators excluded the same three patients due to a compromising abnormality.

### 2.7. Statistical Analysis

Data normality was evaluated with the Shapiro-Wilk test and histogram visualization. Continuous, normally distributed data are reported as mean ± standard deviation (SD) and compared between groups using the* t*-test. Discrete and skewed continuous data are reported as median (interquartile range [IQR]) and compared using the Mann–Whitney *U* test. Categorical data are reported as count (proportion) and compared using Pearson's*χ*^2^ or Fisher's exact test, as appropriate. Confidence intervals for relative proportions of CT-identified osteoporosis and the subset of patients who did not have a diagnosis or medication listed in discharge data were calculated using the thresholds described by Pickhardt et al. [8]. All statistical calculations were performed with Stata/SE 14.1 (StataCorp LP, College Station, TX) using an a priori two-sided significance level of 0.05.

## 3. Results

Of the 252 women ≥ 65 years old who met inclusion criteria, 37 patients were excluded for death within 24 hours of admission (*n* = 5) or inadequate imaging/unusable vertebral bodies at or adjacent to the L1 level (*n* = 32). The remaining 215 comprised the study cohort. Using the threshold described above, 101 women (47%) retrospectively had evidence of osteoporosis by CT scan, leaving 114 (53%) without osteoporosis.

Women with osteoporosis were older (81.4 ± 8.2 versus 77.3 ± 8.3, *p* < 0.001) and more likely to have sustained a ground-level fall (41 [40.6%] versus 29 [25.4%], relative risk [RR] 1.59 [95% CI 1.08–2.36], *p* = 0.02). Demographics and clinical characteristics between osteoporotic and nonosteoporotic groups were otherwise relatively similar (Table 1). Twenty patients died in hospital. There were no differences between groups with regard to any of the following outcomes: hospital length of stay, discharge disposition, inpatient cost, 30-day readmission, 30-day mortality, and 1-year mortality.

Among survivors to discharge, 63 (67.0%) of osteoporotic patients were discharged to a skilled nursing facility (SNF) compared to 55 (54.5%) of nonosteoporotic patients. This difference approached significance on multivariate analysis (RR 1.23 [95% CI 0.98–1.55], *p* = 0.07).

Only 55 (59%) of the 94 patients with CT-identified osteoporosis who survived to discharge had a listed osteoporosis diagnosis and/or corresponding evaluation/treatment plan: 24 had a medication prescribed before their traumatic injury; the other 31 of 55 had a new medication prescribed at time of discharge. The remaining thirty-nine (41%) patients with retrospectively identified osteoporosis did not have a marker for the recognition of osteoporosis by the acute care team. Undiagnosed and untreated osteoporosis proportions did not differ markedly using more sensitive, less specific criteria (Table 2) [8]. Among women with retrospectively identified osteoporosis, the proportion of osteoporosis recognition did not differ between women who sustained a ground-level fall and those with other injury mechanisms (17 [45%] versus 22 [39%], *p* = 0.60).

## 4. Discussion

In this retrospective study, we utilized routine admission CT in an opportunistic fashion to evaluate older women for low L1 BMD. We found that nearly half of those admitted for traumatic injuries had underlying osteoporosis using the most specific of criteria of <110 HU. However, 41% of those women did not have documentation conveying this finding, either in the discharge summary or problem list, or the documentation of medications used to treat osteoporosis. Of the women with evidence of osteoporosis by CT, we found that just 12% were deemed osteoporotic in the problem list or discharge summary.

DXA remains the objective gold standard in BMD assessment (osteoporosis defined as a *T*-score of <−2.5) during routine outpatient care. Despite increased fracture risk and increased mortality in the population with falls, screening for chronic bone loss remains underutilized even in this patient population [1, 12]. It is worth noting that DXA is not reimbursed in the inpatient setting and is largely delegated to outpatient providers. As a result, appropriate follow-up for this chronic disease is susceptible to the communication breakdowns that are common when transitioning from inpatient to outpatient settings [13].

Of the 94 women with evidence of osteoporosis by CT who survived to discharge, only 31 (31%) were prescribed new medications during admission and only 26 (25%) were on previously prescribed medications that could benefit osteoporosis. It is possible that some acute care providers deferred initiation of bone modifying medications, such as bisphosphonates, because of the theoretical concern that these medications impair fracture healing [14, 15]. In order to improve sensitivity, we also included vitamin D and/or calcium as additional surrogates for initiation of osteoporosis treatment. Still, a substantial number of older women with osteoporosis did not receive any medications to promote bone health.

Of note, there were also a number of patients who did not meet the CT-based threshold for osteoporosis but yet had evidence of osteoporosis recognition based on their medication list or diagnosis list (42 [41%] among survivors to discharge). This is likely an effect of the highly specific, but poorly sensitive HU-based threshold of 110. When more sensitive thresholds are considered, more patients are considered osteoporotic (Table 2). However, regardless of threshold used, the estimated proportion of patients with osteoporosis by CT criteria who are discharged without medications or formal diagnoses remains between 41 and 47% in this study cohort.

Osteoporosis evaluation and treatment is largely considered within the purview of primary care: a perspective that may explain the limited evaluation and treatment initiation in an at-risk population during an admission for trauma. Multiple investigations have focused on improving the transition to outpatient care and referral for evaluation of osteoporosis after discharge [16, 17]. These studies have demonstrated improved treatment and evaluation with such methods as a dedicated osteoporosis health professional, fracture liaison nurse, or a letter to the patient's primary care provider. However, diagnosis by CT-derived BMD could streamline initiation of interventions, reduce risk of missed communication, and provide considerable cost savings.

Fragility fractures are a significant public health issue and treatment of osteoporosis has been found to be effective in reducing morbidities, such as secondary fracture prevention [18, 19]. Many of the organizations and countries that have financial responsibility for covered lives have instituted formal protocols for identifying and treating fragility fractures and osteoporosis, ultimately to the benefit of the patient [20, 21]. The ability to utilize existing CT scans to assess osteoporosis could be beneficial for patients and the health care system. Simply providing patients with information regarding their diagnosis of osteoporosis improves the likelihood that a patient will have their osteoporosis addressed by their primary care provider [22].

Opportunistic diagnosis of osteoporosis using CT scans could also serve an unmet need in hospitals that serve as a safety-net, such as ours [23]. By definition, safety-net hospitals serve low income, medically, and socially vulnerable patients regardless of their ability to pay. Economically disadvantaged individuals with chronic conditions have high rates of readmission and emergency department usage following initial hospitalization. Additionally, this population faces greater challenges in receiving pre- and postinjury care [24]. Point-of-care diagnosis could be valuable in the acute care setting, as hospitalization is an opportunity for the patient to be assessed for osteoporosis by CT BMD in a cost- and time-effective manner. Recognition of low BMD as part of trauma care may improve care transitions and lead to efficient arrangement of subsequent interventions and appointments. In a safety-net hospital, CT could also provide an early diagnosis of bone loss in the late-middle age population (55–64 years of age), who do not typically qualify for insurance coverage of outpatient DXA [2, 25].

The present study has several limitations. It is retrospective and excludes patients without imaging, which contributed to a smaller sample size. Participants are exclusively from an ICU population, so severity of injuries is greater than that of a typical population of older adults admitted with trauma. We note that patients admitted to general orthopedic services, especially those with medicine comanagement, are more likely to receive a diagnosis and subsequent plan of care for osteoporosis.

## 5. Conclusion

Trauma patients often undergo routine CT imaging, which provides a unique opportunity to diagnose older women with osteoporosis. Osteoporosis poses a significant risk factor for fractures, future falls, and death. Trauma and other acute care teams should consider using opportunistic imaging to assess older women for osteoporosis, especially those in safety-net settings, and provide a bridge to outpatient services.

## Figures and Tables

**Table 1:** Patient demographics and clinical characteristics.

	No osteoporosis (L1 HU ≥ 110) *N* = 114	Osteoporosis (L1 HU < 110) *N* = 101	*p* value
Age, years	77.3 ± 8.3	81.4 ± 8.2	<0.001
CCI, score	0 (0–2)	1 (0–2)	0.08
Type of injury			0.76
Fall	62 (54.4)	61 (60.4)	
Blunt	49 (43.0)	38 (37.6)	
Penetrating	1 (0.9)	0	
Other	2 (1.8)	2 (2.0)	
Ground-level fall	29 (25.4)	41 (40.6)	0.02
ISS	14 (10–21)	14 (10–18)	0.67
Head AIS			0.51
0	86 (75.4)	82 (81.2)	
1	4 (3.5)	4 (4.0)	
2	24 (21.1)	15 (14.9)	
Received mechanical ventilation	33 (29.0)	23 (22.8)	0.30
ICU LOS, days	2.3 (1.3–4.7)	2.2 (1.5–4.7)	0.75
Hospital LOS, days	6 (4–10)	7 (5–11)	0.20
Disposition			0.21
Home with assist	3 (2.6)	4 (4.0)	
Home	37 (32.5)	25 (24.8)	
Outpatient acute care	1 (0.9)	1 (1.0)	
Rehab	5 (4.4)	1 (1.0)	
SNF	55 (48.3)	63 (62.4)	
In-hospital death	13 (11.4)	7 (6.9)	
Inpatient Cost, $1k	29.3 (17.1–47.7)	29.8 (17.3–51.7)	0.79
Discharged with either osteoporosis diagnosis or medication^a^	42 (41.6)	55 (58.5)	0.02
Preadmission diagnosis/medication	22 (46.8)	26 (45.6)	0.90
New diagnosis/medication	25 (53.2)	31 (54.4)	
Readmission within 30 days^b^	19 (19.8)	18 (19.4)	0.94
30-day mortality^b^	1 (1.0)	3 (3.2)	0.36
1-year mortality^b^	5 (5.2)	8 (8.6)	0.36

Data displayed as *n* (%) for categorical data; mean ± standard deviation for continuous, normally distributed data; and median (interquartile range) for discrete or nonnormally distributed continuous data; HU, Hounsfield unit; CCI, updated Charlson Comorbidity Index; ISS, injury severity score; AIS, abbreviated injury score; ICU, intensive care unit; LOS, length of stay; SNF, skilled nursing facility. ^a^Among the 195 patients who survived to discharge; ^b^among the 189 patients who survived to discharge and have readmission/mortality data available.

**Table 2:** Frequencies and proportion estimates of osteoporosis diagnosis via retrospective CT stratified by diagnostic threshold.

Osteoporosis diagnostic threshold	Patients with osteoporosis by retrospective CT diagnosis		Patients with osteoporosis but without diagnosis or medication in discharge data^a^	
Average HU	*N*	% (95% CI)	*N*	% (95% CI)
<110	94	48 (41–55)	39	41 (32–52)
<135	142	73 (66–79)	66	46 (38–55)
<160	169	87 (81–91)	79	47 (39–54)

CT, computed tomography; HU, Hounsfield unit; CI, confidence interval. ^a^Among the 195 patients who survived to discharge.
